# Multifactorial Evaluation of Color Changes in Material Assemblies Used for Hybrid Abutment Crowns: Role of Ceramics, Surface Treatments, and Resin Cements

**DOI:** 10.1155/ijod/6527246

**Published:** 2026-09-08

**Authors:** Stefani Eken, Mehmet Akça, Gülsüm Sayın Özel, Işıl Karaokutan

**Affiliations:** ^1^ Institute of Health Sciences, Prosthodontics PhD Program, Istanbul Medipol University, Istanbul, Türkiye, medipol.edu.tr; ^2^ Department of Prosthetic Dentistry, School of Dentistry, Medipol Mega University Hospital, Istanbul Medipol University, Istanbul, Türkiye, medipol.edu.tr; ^3^ Department of Prosthetic Dentistry, School of Dentistry, Pamukkale University, Denizli, Türkiye, pau.edu.tr

**Keywords:** color stability, hybrid abutments, surface treatment, thermal aging, zirconia-reinforced lithium disilicate

## Abstract

**Purpose:**

This study aimed to evaluate the individual and combined effects of titanium surface treatment (ST), ceramic material (CM), and resin cement (RC) on the color stability of material assemblies used for hybrid abutment crowns following artificial aging.

**Materials and Methods:**

Titanium discs (*n* = 160; 10 mm × 3 mm) were produced and allocated to four ST protocols: untreated control, 50 µm Al_2_O_3_ sandblasting, 110 µm Al_2_O_3_ sandblasting, and tribochemical silica coating (CoJet). Ceramic components were fabricated using lithium disilicate (LS) and zirconia‐reinforced lithium disilicate (ZLS) ceramics and cemented with either an opaque self‐curing RC (Multilink Hybrid Abutment Cement) or an opaque dual‐curing RC (Panavia V5). Color differences (Δ*E*_00_) were measured using a spectrophotometer before and after 5000 thermocycles. Group differences were analyzed using the Kruskal–Wallis and Mann–Whitney *U* tests. To evaluate the independent and interactive effects of ST, CM, and RC, a three‐way factorial analysis of variance (ANOVA) was performed.

**Results:**

Significant differences in Δ*E*_00_ values were observed among the experimental groups (*p* < 0.001). The greatest color change was recorded in ZLS specimens bonded with self‐curing RC to 50 µm Al_2_O_3_‐treated titanium surfaces, whereas the lowest color change was observed in specimens bonded with dual‐curing RC to untreated titanium surfaces. Three‐way ANOVA demonstrated that ST had a significant main effect on overall color change (*p* < 0.001, *ηp* ^2^ = 0.362), whereas CM and RC showed no significant independent effects (*p* > 0.05). However, significant interactions between surface treatment and CM, surface treatment and RC, and the three‐way interaction indicated that the influence of ceramic type and cement system on color stability depended on the titanium ST.

**Conclusion:**

ST demonstrated the largest independent effect on the color stability of material assemblies used for hybrid abutment crowns after artificial aging. However, the final optical outcome was also influenced by significant interactions among ST, CM, and RC.

## 1. Introduction

Recent advancements in dentistry have significantly expanded the scope of implantology, providing patients with effective options to restore both the esthetics and function [1]. In contemporary clinical practice, influenced by the growing impact of social media and advertising, esthetics have emerged as a primary concern for many patients [2]. As a result, the selection of appropriate materials for both abutments and crowns has become critical in achieving optimal esthetic and functional outcomes [3].

Titanium alloy (Ti‐6Al‐4V) remains the most widely used material for implant abutments due to its favorable mechanical properties and biocompatibility [4, 5]. However, its inherent gray color may pose esthetic challenges, particularly in patients with thin gingival biotypes or translucent soft tissues, thereby compromising the final esthetic result [6, 7]. Several studies have emphasized that the grayish reflection of the titanium base (Ti‐base) may negatively affect the final shade of ceramic restorations, even when high‐translucency ceramics are used. This phenomenon is especially critical in the anterior region, where a minimal color mismatch can lead to patient dissatisfaction [8].

To address such limitations, one‐piece zirconia abutments have been introduced as a more esthetic alternative. Nevertheless, their clinical application is limited by concerns related to wear and the risk of fracture at the implant–abutment interface [9, 10]. To overcome these mechanical shortcomings, two‐piece systems—such as hybrid abutments or hybrid abutment crowns—have been developed, typically involving the combination of a ceramic component with a titanium base [11, 12].

In these systems, a custom abutment is fabricated by bonding a ceramic substructure (e.g., zirconia or lithium disilicate [LS]) to a prefabricated titanium base. A full‐ceramic crown is then cemented either directly onto the titanium base or onto this customized abutment [13]. These esthetic ceramic materials (CMs) enhance the appearance of the final restoration. However, as these restorations involve bonding between dissimilar materials, the strength and durability of the adhesive interface are critical for long‐term clinical success.

The bonding mechanism relies on a combination of micromechanical and chemical interactions [14]. Sandblasting with aluminum oxide (Al_2_O_3_) particles is a commonly used surface treatment that enhances mechanical retention by increasing the surface area and wettability while reducing surface tension [15]. Chemical bonding is facilitated using metal primers containing functional monomers, such as MDP or 4‐META, which form covalent bonds with metal oxides [16–18]. Another effective method is tribochemical silica coating—employed in systems such as CoJet and Rocatec—which provides both micromechanical interlocking and chemical bonding [19].

Resin cements (RCs) are the most frequently used luting agents in these systems. Among them, self‐cure and dual‐cure cements are often preferred, particularly in clinical situations where light penetration may be insufficient due to restoration thickness [20]. However, inadequate bonding may lead to microleakage and cement discoloration over time, potentially compromising the restoration’s color stability [21–23].

Color differences in dentistry are commonly assessed using the CIE Lab ^∗^ system, in which the overall color change is expressed as Δ*E* [24]. A Δ*E* value represents the degree of perceptible difference between two colors, and thresholds have been proposed to define clinical acceptability. Generally, Δ*E* values below 1.0 are considered imperceptible to the human eye, while values up to 3.3 are regarded as clinically acceptable in dental restorations [25]. Therefore, evaluating Δ*E* after artificial aging provides meaningful insight into the long‐term esthetic performance of restorative materials.

Although numerous studies have investigated the effects of CM type, cement type, and surface conditioning on the strength of adhesion [26, 27], limited data are available regarding their impact on color stability, particularly under artificially accelerated aging conditions. An evaluation was conducted to assess how different CMs, RCs, and surface treatments affect the color stability of material assemblies used for hybrid abutment crowns following aging.

It is hypothesized that variations in ceramic systems, luting cements, and surface treatments do not significantly influence the color stability of material assemblies used for hybrid abutment crowns subjected to artificial aging.

## 2. Materials and Methods

The experimental model was designed to simulate the titanium–cement–ceramic complex of a hybrid abutment crown system.

Titanium discs (*N* = 160) were fabricated using CAD/CAM procedures. The titanium alloy specimens were separate into four groups according to their surface conditioning (*n* = 40). Each group was further divided into two sub‐groups based on CM and RCs. A total of 16 different subgroups were produced (*n* = 10). Table 1 presents the CMs and RCs used throughout the study, and Table 2 shows the experimental design of the study.

**Table 1:** Ceramic systems and resin cements employed in the study.

Material type	Brand (code)	Composition (main ingredients)	Manufacturer
Lithium disilicate glass ceramic	IPS e.max CAD (LS)	58%–80% SiO_2_,11%–19% Li_2_O, 0%–13% K_2_O,0%–8% ZrO_2_, 0%–5% Al_2_O_3_	Ivoclar Vivadent, Schaan, Liechtenstein
Zirconia‐reinforced Lithium silicate	Celtra Duo PC/FC (ZLS)	58%SiO_2_, lithium metasilicate, disilicate and phosphate crystals,10% zirconia crystals, plus minor additives	Dentsply Sirona, Konstanz, Germany
Self‐curing adhesive resin cement	Multilink Hybrid Abutment (SC)	Dimethacrylate, HEMA, barium glass, ytterbium trifluoride, spheroid mixed oxide, titanium oxide	Ivoclar Vivadent, Schaan, Liechtenstein
Dual‐curing adhesive resin cement	Panavia V5 (DC)	Bis‐GMA, TEGDMA, hydrophobic aromatic and hydrophilic aliphatic dimethacrylate, initiators, accelerators, silanated glass fillers, colloidal silica, pigments	Kuraray Noritake Dental Inc., Okayama, Japan

**Table 2:** Experimental design of the study according to titanium surface treatment, ceramic material, and resin cement.

Titanium surface treatment (ST)	Ceramic material (CM)	Resin cement (RC)
Control	Lithium disilicate (LS)	Multilink Hybrid Abutment
	Lithium disilicate (LS)	Panavia V5
	Zirconia‐reinforced lithium disilicate (ZLS)	Multilink Hybrid Abutment
	Zirconia‐reinforced lithium disilicate (ZLS)	Panavia V5
50 µm Al_2_O_3_ sandblasting	Lithium disilicate (LS)	Multilink Hybrid Abutment
	Lithium disilicate (LS)	Panavia V5
	Zirconia‐reinforced lithium disilicate (ZLS)	Multilink Hybrid Abutment
	Zirconia‐reinforced lithium disilicate (ZLS)	Panavia V5
110 µm Al_2_O_3_ sandblasting	Lithium disilicate (LS)	Multilink Hybrid Abutment
	Lithium disilicate (LS)	Panavia V5
	Zirconia‐reinforced lithium disilicate (ZLS)	Multilink Hybrid Abutment
	Zirconia‐reinforced lithium disilicate (ZLS)	Panavia V5
Tribochemical silica coating (CoJet)	Lithium disilicate (LS)	Multilink Hybrid Abutment
	Lithium disilicate (LS)	Panavia V5
	Zirconia‐reinforced lithium disilicate (ZLS)	Multilink Hybrid Abutment
	Zirconia‐reinforced lithium disilicate (ZLS)	Panavia V5

*Note:* Experimental design of the study showing the combinations of titanium surface treatment (ST), ceramic material (CM), and resin cement (RC) used to simulate the hybrid abutment crown system. The study followed a balanced 4 × 2 × 2 factorial design, resulting in 16 experimental groups (*n* = 10 per group).

### 2.1. Preparation of Titanium Samples

One hundred and sixty titanium discs (10 × 3 mm) were manufactured with a CNC milling system (Premium 5030, Eiterfeld, Germany). After production, each specimen was mounted in chemically cured acrylic resin (Meliodent, Heraeus Kulzer, South Bend, USA) and cleaned in an ultrasonic cleaner for 5 min. The specimens were then randomly assigned into four groups based on the surface treatment to be applied.

In the first group, no surface modification was performed, serving as the control group. The second group underwent sandblasting with 50 μm aluminum oxide (Al_2_O_3_) particles at a 45° angle and 2 bar pressure for 10 s. Similarly, the third group was treated under the same conditions but with 110 μm Al_2_O_3_ particles. For the fourth group, a tribochemical silica coating was performed with 30 μm silica‐modified Al_2_O_3_ particles (CoJet, 3M ESPE, Germany) at 2 bar pressure for 15 s.

Following these procedures, all specimens were subjected to a secondary cleaning process in 10% alcohol using an ultrasonic cleaner for 180 s.

### 2.2. Preparation of Ceramic Samples

Following surface treatment, each group was furthermore separate into two subgroups for bonding with ceramic specimens (*n* = 10 per subgroup). The ceramic types used are as follows:

1. LS ceramic (IPS E.max CAD (HT, A1), Ivoclar Vivadent AG, Liechtenstein) and
2. Zirconia‐reinforced lithium silicate (ZLS) ceramic (Celtra Duo (HT, A1), Dentsply Sirona, Germany).

Ceramic samples were fractured from prefabricated blocks into cylindrical shapes with a 5 mm diameter and 3 mm height. Following milling, IPS e.max CAD specimens underwent crystallization firing according to the manufacturer’s instructions using a ceramic furnace (Programat P310, Ivoclar Vivadent, Schaan, Liechtenstein). Celtra Duo specimens were fired according to the manufacturer’s recommendations. After the respective firing procedures, all ceramic specimens were mechanically polished according to the manufacturers’ recommendations. No glazing procedure was performed. Both CMs received the same final surface finishing protocol prior to cementation and color measurements.

### 2.3. Cementation of Ceramic and Titanium Samples

Zirconia‐reinforced lithium silicate specimens were conditioned with 4.8% hydrofluoric acid gel (IPS Ceramic Etching Gel, Ivoclar Vivadent, Liechtenstein) for 30 s, while LS samples were treated with the same etching agent for 20 s. Following the etching procedure, all samples were thoroughly rinsed with water and dried.

Ceramic samples are divided into two according to the type of RC

• Multilink Hybrid Abutment Cement (opaque shade; Ivoclar Vivadent, Liechtenstein).
• Panavia V5 Cement (opaque shade; Kuraray Noritake Dental, Japan).

Monobond plus was applied to the ceramic and titanium surfaces of the groups, bonded with Multilink Hybrid Abutment cement for 60 s, and dried. Clearfil Ceramic Primer Plus was applied to the ceramic and titanium surfaces of the groups, bonded with Panavia V5 cement, and dried. RC was applied to the ceramic surface and bonded to the titanium surface with the Gilmore device under 15 N pressure. Specimens bonded using Panavia V5 RC were polymerized with a light‐emitting diode (LED) unit for 5 s (VALO™ Cordless, Ultradent, South Jordan, USA). For the samples cemented with Multilink Hybrid Abutment cement, autopolymerization was allowed to proceed for 7 min without light activation. Marginal excess cement was carefully removed with a scalpel. To minimize variability, all cementation procedures were performed by a single operator.

### 2.4. Thermal Aging and Color Measurement

All specimens were stored in a humid environment at 37 °C for 1 day (24 h) and subsequently subjected to thermocycling for 5000 thermocycles between 5 and 55°C, with a dwell time of 20 s in distilled water at each temperature. Thermocycling was performed as an artificial aging method to simulate the thermal stresses encountered in the oral environment.

Color evaluations were performed using a digital spectrophotometer (Vita Easyshade™ V, Vita Zahnfabrik, Bad Säckingen, Germany). Prior to the measurement, the device was calibrated in accordance with the manufacturer’s guidelines.

All measurements were performed by a single operator against a standardized black background. Titanium specimens were embedded in acrylic resin blocks, allowing repeated measurements to be obtained from the same location and orientation throughout the study. Since the spectrophotometer incorporates its own calibrated light source, measurements were performed under standardized laboratory conditions.

For each specimen, three consecutive measurements were obtained and averaged to determine the *L*  ^∗^, *a*  ^∗^, and *b*  ^∗^ values. Measurements were performed before and after thermocycling. The color difference (Δ*E*_00_) was calculated using the CIEDE2000 formula as the difference between the pre‐aging and post‐aging measurements of the same specimen. The obtained Δ*E*_00_ values were used to evaluate the effect of thermal aging on the color stability of the tested restorative systems.

### 2.5. Statistical Analysis

All statistical analyses were performed using NCSS 2007 software (Number Cruncher Statistical System, Kaysville, UT, USA). Initially, differences in Δ*E*_00_ values among the experimental groups were evaluated using the Kruskal–Wallis test, followed by pairwise comparisons with the Mann–Whitney *U* test, where appropriate. To further investigate the independent and interaction effects of titanium surface treatment (ST), CM, and RC on the color parameters (Δ*E*_00_, *L*  ^∗^, *a*  ^∗^, and *b*  ^∗^), a three‐way factorial analysis of variance (ANOVA) was performed. Prior to ANOVA, the normality of model residuals was assessed using the Shapiro–Wilk test, and homogeneity of variances was evaluated using Levene’s test. Although minor deviations from these assumptions were observed, the balanced 4 × 2 × 2 factorial design with equal sample sizes in all experimental groups supported the use of factorial ANOVA. Effect sizes were expressed as partial eta‐squared (*ηp* ^2^). Statistical significance was set at *p* < 0.05.

## 3. Results

**Figure 1:**
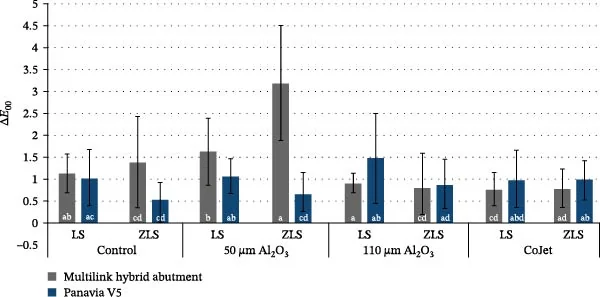
Mean Δ*E*_00_ values for each group. Different superscript letters within the same column indicate a significant difference between groups (Mann–Whitney *U* test, *p* < 0.05).

The Δ*E*_00_ values for all experimental groups are presented in Table 3, and the corresponding mean values are illustrated in Figure 1. The Kruskal–Wallis test demonstrated a significant difference in Δ*E*_00_ values among the experimental groups (*p* = 0.001). Pairwise comparisons were subsequently performed using the Mann–Whitney *U* test, and statistically significant differences are indicated by the different superscript letters in Figure 1.

**Table 3:** Color difference (Δ*E*_00_) values according to titanium surface treatment, ceramic material, and resin cement.

Titanium surface treatment (ST)	Ceramic material (CM)	Resin cement (RC)	*n*	Δ*E*_00_ (Mean ± SD)	Min–Max (median)
Control	LS	Multilink Hybrid Abutment	10	1.11 ± 0.44	0.55–1.76 (1.00)
	LS	Panavia V5	10	1.01 ± 0.65	0.49–2.60 (0.83)
	ZLS	Multilink Hybrid Abutment	10	1.37 ± 1.04	0.29–3.24 (1.16)
	ZLS	Panavia V5	10	0.52 ± 0.37	0.22–1.39 (0.37)
50 µm Al_2_O_3_ sandblasting	LS	Multilink Hybrid Abutment	10	1.61 ± 0.76	0.70–2.71 (1.36)
	LS	Panavia V5	10	1.04 ± 0.37	0.52–1.52 (1.07)
	ZLS	Multilink Hybrid Abutment	10	3.17 ± 1.30	1.05–4.40 (3.47)
	ZLS	Panavia V5	10	0.63 ± 0.50	0.28–1.98 (0.54)
110 µm Al_2_O_3_ sandblasting	LS	Multilink Hybrid Abutment	10	0.88 ± 0.21	0.64–1.35 (0.86)
	LS	Panavia V5	10	1.45 ± 1.06	0.57–3.10 (0.79)
	ZLS	Multilink Hybrid Abutment	10	0.78 ± 0.80	0.23–2.28 (0.42)
	ZLS	Panavia V5	10	0.84 ± 0.58	0.12–2.17 (0.84)
Tribochemical silica coating (CoJet)	LS	Multilink Hybrid Abutment	10	0.74 ± 0.39	0.12–1.55 (0.76)
	LS	Panavia V5	10	0.97 ± 0.67	0.15–1.99 (0.96)
	ZLS	Multilink Hybrid Abutment	10	0.77 ± 0.44	0.21–1.49 (0.89)
	ZLS	Panavia V5	10	0.98 ± 0.38	0.49–1.67 (0.94)

*Note:* Mean color difference (Δ*E*_00_) values of the experimental groups according to titanium surface treatment (ST), ceramic material (CM), and resin cement (RC). Values are presented as mean ± standard deviation (SD), together with minimum–maximum values and medians. Overall group comparisons were performed using the Kruskal–Wallis test (*p* = 0.001).

The highest color change (Δ*E*_00_ = 3.17 ± 1.30) was observed in ZLS specimens cemented with Multilink Hybrid Abutment following 50 µm Al_2_O_3_ sandblasting of the titanium surface. In contrast, the lowest color change (Δ*E*_00_ = 0.52 ± 0.37) was observed in ZLS specimens cemented with Panavia V5 on untreated titanium surfaces. Overall, color stability varied considerably among the different combinations of ST, CM, and RC.

To further investigate the independent and interaction effects of the three experimental factors, a three‐way factorial ANOVA was performed.

For overall color change (Δ*E*_00_), ST demonstrated a significant main effect (*p* < 0.001, *ηp* ^2^ = 0.362), whereas CM (*p* = 0.745) and RC (*p* = 0.857) did not show significant independent main effects. However, significant interaction effects were identified between ST and CM (*p* < 0.001, *ηp* ^2^ = 0.114), ST and RC (*p* < 0.001, *ηp* ^2^ = 0.260), and among all three investigated factors (*p* = 0.014, *ηp* ^2^ = 0.071). No significant interaction was observed between CM and RC (*p* = 0.244).

Regarding the individual CIELAB color coordinates, *L*  ^∗^ was significantly influenced by ST (*p* < 0.001), CM (*p* = 0.044), and RC (*p* < 0.05). In addition, all two‐way interactions and the three‐way interaction were statistically significant (*p* < 0.05).

For the *a*  ^∗^ coordinate, significant main effects were observed for CM (*p* < 0.001) and RC (*p* < 0.001), whereas ST did not significantly influence *a*  ^∗^ values (*p* = 0.070). Among the interaction terms, only the interaction between CM and RC reached statistical significance (*p* = 0.010).

For the *b*  ^∗^ coordinate, significant main effects were identified for ST (*p* = 0.008), CM (*p* < 0.001), and RC (*p* < 0.001). Furthermore, all two‐way interactions and the three‐way interaction were statistically significant (*p* < 0.05). Among all investigated effects, CM exhibited the largest effect size for *b*  ^∗^ (*ηp* ^2^ = 0.439).

Overall, the factorial analysis demonstrated that color‐related outcomes were influenced not only by the independent effects of the investigated variables but also by their interactions. While ST was the dominant factor of overall color change (Δ*E*_00_), the individual CIELAB coordinates exhibited distinct response patterns, indicating that the optical behavior of hybrid abutment restorations is governed by multifactorial interactions among ST, CM, and RC.

## 4. Discussion

The present study evaluated the individual and combined effects of ST, CM, and RC on the color stability of material assemblies used for hybrid abutment crowns after artificial aging. The null hypothesis was partially rejected as the investigated factors and their interactions significantly influenced the color‐related outcomes. The three‐way factorial analysis demonstrated that overall color stability cannot be explained by a single restorative variable alone but rather by the interaction among ST, CM, and RC.

Hybrid abutment crown systems have become increasingly popular because they combine the favorable mechanical properties of titanium with the esthetic advantages of ceramic restorations [12, 28]. Previous studies have reported mechanical complications associated with one‐piece zirconia abutments, particularly fractures occurring in the cervical region [29–31]. Consequently, titanium‐based systems have been proposed as a more reliable alternative while maintaining acceptable esthetic outcomes [32]. The use of titanium‐based restorations, therefore, necessitates a better understanding of the factors that may influence long‐term color stability.

One notable finding of the present study was the magnitude of the color change observed in certain experimental groups following thermocycling. Although all specimens consisted of relatively thick ceramic discs (3 mm), Δ*E*_00_ values reached 3.17 in one experimental group. Such color alterations may initially appear unexpectedly high, considering the thickness of the CM. However, thermocycling subjects the ceramic, RC, and titanium components to repeated thermal expansion and contraction. Because these materials possess different coefficients of thermal expansion and different water sorption characteristics, thermal aging may induce interfacial stresses, water uptake within the RC layer, and subtle changes in light transmission through the restorative complex [33]. Previous studies have also demonstrated a close relationship between water sorption and color change in RCs following artificial aging [34]. Therefore, these processes could potentially contribute to optical alterations even in the absence of visible structural degradation. Furthermore, color measurements in hybrid restorations represent the cumulative optical response of multiple interacting layers rather than the behavior of a single material [35]. Nevertheless, interfacial stress, water uptake, and changes in light transmission were not directly evaluated in the present study and should therefore be regarded as literature‐supported explanations rather than mechanisms demonstrated by the present findings.

ST demonstrated the largest independent effect on the overall color change, exhibiting the largest effect size among all investigated main effects. However, the significant interaction effects showed that the final optical outcome also depended on the CM and RC. Surface conditioning procedures such as airborne particle abrasion and tribochemical silica coating are traditionally applied to improve micromechanical retention and enhance bonding between titanium and RC [36–38]. Previous studies have demonstrated that titanium substrates can influence the optical behavior of ceramic restorations by reducing lightness and chroma because of their metallic optical characteristics [39]. The differences observed among the surface treatment protocols in the present study suggest that modification of the titanium surface may also be associated with color stability following thermocycling. Surface treatments modify the topography and surface energy of titanium and may alter the way light is reflected, scattered, and transmitted through the titanium–cement–ceramic assembly [39]. These optical modifications may become more pronounced following thermal aging, thereby contributing to differences in color stability among the investigated surface treatment protocols. However, because surface roughness, surface topography, and optical reflection were not directly characterized, the mechanisms responsible for the differences among the surface treatment protocols cannot be established from the present findings. Thus, ST demonstrated the largest independent effect within the present experimental model, while the overall color outcome remained dependent on its interactions with the CM and RC.

The significant interaction between ST and RC provides further insight into the mechanisms underlying color stability. Although RC did not demonstrate a significant independent effect on overall Δ*E*_00_ values, its influence depended on the ST applied. Similarly, previous studies have shown that the esthetic and clinical performance of RCs should be interpreted within the restorative system rather than as an isolated material. Oancea et al. reported that the influence of cement shade became more evident only in specific ceramic–substrate combinations, while a recent study concluded that the long‐term performance of Ti‐base restorations depends on the combined effects of the CM, surface pretreatment, and cementation protocol [39, 40]. Panavia V5 contains the functional monomer 10‐MDP, which enhances chemical bonding to metal oxides and zirconia; however, the present findings indicate that stronger chemical adhesion alone does not necessarily ensure greater color stability [41, 42]. Instead, the long‐term optical behavior of hybrid abutment restorations appears to be governed by the interaction between titanium surface characteristics, CM, and RC.

Although the CM did not demonstrate a significant independent effect on overall Δ*E*_00_ values in the three‐way factorial ANOVA, significant interaction effects indicated that its influence depended on the accompanying ST and RC. Similar observations have been reported in recent clinical studies demonstrating comparable clinical performance of LS‐ and zirconia‐based restorations bonded to Ti‐bases, whereas laboratory investigations have suggested differences in their behavior following artificial aging [43]. These apparently conflicting findings indicate that the influence of the CM depends on the restorative system and the experimental conditions rather than on the ceramic itself. In the present study, the significant interaction effects support this interpretation, indicating that the contribution of the CM to color stability should be considered together with ST and RC.

Beyond the overall color difference (Δ*E*_00_), the individual CIELAB color coordinates demonstrated distinct response patterns, reflecting the multifactorial nature of the optical behavior in hybrid abutment restorations. Lightness (*L*  ^∗^) was influenced by all three investigated factors and their interactions, indicating that brightness is particularly sensitive to variations in ST, CM, and RC. In contrast, the *a*  ^∗^ coordinate was predominantly affected by CM and RC, whereas the *b*  ^∗^ coordinate showed the greatest dependence on CM, exhibiting the largest effect size among all investigated variables. These findings are consistent with previous reports demonstrating that the optical performance of implant‐supported ceramic restorations is influenced by multiple restorative components, including the abutment material, ceramic characteristics, and luting procedures [40, 44, 45]. Kim and Kim reported that the masking ability of ceramic restorations over titanium is strongly associated with optical interactions between the ceramic and the underlying substrate, whereas Xinran et al. and Gehrke et al. demonstrated that both cement selection and restorative material characteristics contribute to the final color of titanium‐based ceramic restorations [8, 40, 44]. Likewise, Elter and Tak [45] emphasized that ST should be considered together with ceramic thickness and cement characteristics when optimizing the final esthetic outcome. Collectively, these observations support the present findings and highlight that evaluation of the individual CIELAB coordinates, in addition to Δ*E*_00_, provides a more comprehensive understanding of the mechanisms underlying color changes following artificial aging.

From a clinical perspective, optimization of esthetic outcomes should not rely on selecting a single restorative material. Instead, ST, CM, and RC should be selected as an integrated restorative system because their combined behavior determines the final optical outcome, particularly in esthetically demanding regions.

The findings of this study should be interpreted considering several limitations. Although ceramic specimens were fabricated using standardized CAD/CAM dimensions, their final thicknesses were not remeasured after crystallization and polishing. Likewise, the cement film thickness was standardized using a controlled cementation protocol but was not directly quantified. An important limitation of the present study was the absence of quantitative characterization of the titanium surfaces after treatment. Surface roughness and topography were not evaluated using profilometry or microscopic analysis. Therefore, although significant differences were observed among the surface treatment protocols, these differences cannot be directly attributed to specific roughness or topographical characteristics. Furthermore, the use of disc‐shaped specimens facilitated standardized spectrophotometric measurements but did not completely reproduce the anatomical geometry of clinical hybrid abutment restorations. Finally, artificial aging was limited to thermocycling without cyclic mechanical loading. Future studies should combine optical analysis with quantitative surface characterization, bond strength evaluation, and long‐term thermo‐mechanical aging to further elucidate the interaction between ST, CM, and RC under clinically relevant conditions.

## 5. Conclusion

Within the limitations of this in vitro study, ST demonstrated the largest independent effect on the overall color stability of material assemblies used for hybrid abutment crowns after artificial aging. However, the significant interaction effects indicated that the final optical outcome was not determined by ST alone but depended on its interactions with the CM and RC. Therefore, ST, CM, and RC should be considered as components of an integrated restorative system rather than as independent determinants of the esthetic performance.

## Author Contributions

**Gülsüm Sayın Özel:** conceptualization, methodology, supervision. **Mehmet Akça:** methodology. **Işıl Karaokutan:** formal analysis, investigation. **Stefani Eken:** data curation, visualization, writing – original draft, writing – review and editing.

## Funding

This study did not receive any specific funding.

## Disclosure

All authors have read and approved the final version of the manuscript. The corresponding author had full access to all of the data in this study and takes complete responsibility for the integrity of the data and the accuracy of the data analysis.

## Ethics Statement

This in vitro study did not involve human participants or animals. Therefore, ethical approval was not required according to the institutional regulations.

## Consent

The authors have nothing to report.

## Conflicts of Interest

The authors declare no conflicts of interest.

## Data Availability

The data supporting the findings of this study are available from the corresponding author upon reasonable request.
